# Optical Sensor of Thermal Gas Flow Based on Fiber Bragg Grating

**DOI:** 10.3390/s17020374

**Published:** 2017-02-15

**Authors:** Xu Jiang, Keda Wang, Junqing Li, Hui Zhan, Zhenan Song, Guohang Che, Guohui Lyu

**Affiliations:** 1 College of Information Science and Technology, Heilongjiang University, Harbin 150080, China; 1995042@hlju.edu.cn; 2 Research Center for Fiber Optic Sensing Technology National Local Joint Engineering, Electronic Engineering College, Heilongjiang University, Harbin 150080, China; 2151243@s.hlju.edu.cn (K.W.); 2000092@hlju.edu.cn (H.Z.); 2131232@s.hlju.edu.cn (Z.S.); 2131242@s.hlju.edu.cn (G.C.); 3 Physics Department, Harbin Institute of Technology, Harbin 150001, China; jqli@hit.edu.cn

**Keywords:** fiber Bragg grating, heat flow sensor, constant power method, light absorbing coating

## Abstract

This paper aims at solving the problem of explosion proof in measurement of thermal gas flow using electronic sensor by presenting a new type of flow sensor by optical fiber heating. A measuring unit based on fiber Bragg grating (FBG) for fluid temperature and a unit for heat dissipation are designed to replace the traditional electronic sensors. The light in C band from the amplified spontaneous emission (ASE) light source is split, with one part used to heat the absorbing coating and the other part used in the signal processing unit. In the heating unit, an absorbing coating is introduced to replace the traditional resistance heating module to minimize the risk of explosion. The measurement results demonstrate a fine consistency between the flow and temperature difference in simulation. The method to enhance the measurement resolution of flow is also discussed.

## 1. Introduction

Measurement of gas flow is a key point in the field of gas application. In recent decades, thermal mass flow (TMF) meters are widely used in measuring the mass of gas [1]. According to the different test principle, TMF meters can be divided into two types: the thermal-distributed one and thermal-dissipated one [2]. While on the basis of difference in detected variables, TMF meters can also be classified into two types, i.e., one type with constant power and the other type with constant temperature difference [3]. The TMF meters have many advantages such as wide applicable field (it can be applied to many kinds of pipeline and different type of gases) [4,5], wide measurement range, and high measurement accuracy and repeatability [6]. 

In the traditional gas metering, gas flow sensors with electric heating are often used. Usually, in the temperature detecting part, the thermal resistance is adopted for the heat flow meter. It is necessary to carefully design the explosion proof mechanism, especially in electric heating part, to prevent the short circuit or electric spark, which is extremely likely to cause catastrophe to the users. It should be pointed out that, in recent years, the gas industry also demands novel techniques to replace the traditional electric heat flow meter in order to reduce the hidden danger. 

The sensor based on optical fiber and its derived components, for example, the fiber Bragg grating (FBG), has many advantages over conventional electrical sensors [7], such as no electrical measurement, anti-electromagnetic interference, intrinsic safety, and high sensitivity. It can easily realize multi-parameter measurement and is very suitable for measuring the temperature and pressure on the occasions with inflammable and explosive gas. For the fiber-based heat flow measurements, the structure design for heat dissipation in a sensor is the challenging aspect. It should be pointed out that Cheng et al. investigated fiber-optic thermal anemometer based on metal-coated fiber Bragg grating [8], in which a 1480 nm LD is used to heat the fiber grating with the Ag coating. In their work, the heating light is introduce into the cladding layer and directly interacts with the metal coating, converting the light energy to the heat energy in the metal coating. In fact, there is another scheme using 980 nm pump heating, since the loss of 980 nm light is larger in single mode fiber, it is not suitable for the long distance transmission of light energy and sensing.

In this paper, starting from considering the consistence of the wavelength of the heating part and the detecting part and the low loss, a sensor of heat flow based on FBG is proposed, in which an ASE light source in the C band is employed and the special absorbing coating is carefully designed without introducing the technique of dislocation welding. The simulation shows that the sensor can achieve all optical measurement and is also explosion-proof with high precision.

## 2. Principle

### 2.1. Scheme of Measurement 

**Figure 1:**
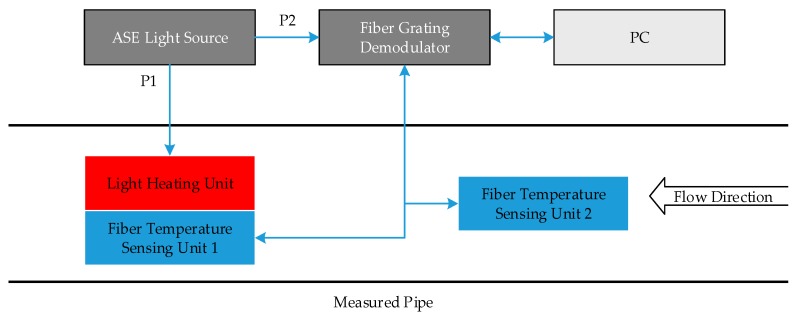
Overall design of the optical sensor of gas flow.

In this paper, an optical fiber sensor of gas flow is presented, which works by measuring the temperature difference. Figure 1 gives the schematic principle of measurement, where two FBG-based temperature sensing units and a light heating unit are integrated in the measured pipe. In order to operate conveniently, one temperature sensing unit and one light heating unit are intentionally designed together. The ASE light source outputs constant power, in which 80% of the total power (labeled by P1) is provided to the light heating unit and the rest power (labeled by P2) is provided to the fiber temperature sensing units 1 and 2. Through the fiber grating demodulator, the temperature difference from two FBG sensors may be monitored in real time. When there is no gas flow in the pipe, the temperature difference between the two FBG temperature sensors is greatest. When there is a gas flow in the pipe, it begins to take heat away from the heating unit, which leads to a temperature decrease in the heating unit. In a short time, the temperature ceases decreasing and trends to a constant as the heating power and the flow are steady. Therefore, the temperature difference between two temperature sensors reaches a stable value smaller than that in the case when there is no flow. The greater the gas flow, the smaller the difference. By measuring the temperature difference, the flow in the pipe may be obtained. The relations between the flow and temperature difference will be clarified in the next section. 

### 2.2. Principle of Measurement

The sensor is based on the principle of convection of heat transfer using a metal sensing head. When measuring, the sensor head is placed in the gas to be measured, there will be natural-convection heat transfer and forced air heat transfer between the sensor head and gas. 

There are many factors related to heat transfer, the most critical one is flow velocity of gas to be measured. If other factors are fixed, the convective heat transfer is a function of the flow velocity. 

When the sensing head which is covered by a metal is heated in flowing gas, between the metal and the gas, the heat exchange is carried out in three ways: conduction, radiation, and convection. Under ideal conditions, this environment is an enclosed thermodynamic system, the heat balance equation of this system can be listed as

(1) $$H=Q_{c}+Q_{k}+Q_{f}$$

where H means heat; $Q_{c}$, $Q_{k}$ and $Q_{f}$ represent heat of convective transfer, conducted heat and radiant heat, respectively. Normally, the temperature difference between the heated metal sensing head and the gas to be measured is less than 300 °C. In this case, the effect of heat radiation $Q_{f}$ on the overall heat is very small, which can be ignored. In order to improve the accuracy, in the real design and manufacture of sensing head, we will reduce its conducted heat $Q_{k}$ to the minimum. 

If the flow velocity is adequate, the effect of natural convection can also be neglected. Therefore, the heat loss of the sensing head is mainly determined by forced convection, that is $H=Q_{c}$. On the basis of Newton cooling formula,

(2) H=A⋅ΔT⋅h=2πld⋅ΔT⋅h

where h expresses heat transfer coefficient; A, l, and d represent the area of sensing head contacting gas (a cylinder), the length and diameter of sensing head, respectively. ΔT means the temperature difference between the sensing head and the gas. 

Correspondingly, determining the heat transfer coefficient h of the metal sensing head, the heat balanced relation between the sensing head and the gas can be ensured. According to the principle of heat conduction, we introduce the following parameters:

$$\text{the Nusselt number}:\;N_{u}=\frac{hd}{\lambda_{f}};$$

$$\text{the Prandt number}:\;P_{r}=\frac{\eta c_{p}}{\lambda_{f}};$$

$$\text{the Reynolds number}:\;R_{e}=\frac{\rho Ud}{\eta }$$

where $\lambda_{f}$, η, and $c_{p}$ represent thermal conductivity of gas to be measured, dynamic viscosity, and the gas specific heat capacity at constant pressure, respectively. ρ denotes the density of the gas, and U is the flow velocity of the gas. All above numbers are dimensionless parameters. The relationship among the heat transfer coefficient, the gas velocity and the physical property parameters can be expressed as a function:

(3) $$N_{u}=f(R_{e},P_{r}).$$

Therefore, the heat balance relation of metal sensing head and gas can be expressed as

(4) $$H=\pi l\lambda_{f}f\left(R_{e},P_{r}\right)\Delta T.$$

To determine, however, the specific relationship between the flow and heat, the key is to get explicit function expression of $N_{u}=f(R_{e},P_{r})$. Based on the theory of King’s Law [9], convection heat transfer equation can be expressed as

(5) $$N_{u}=A_{k}+B_{k}\cdot {R_{e}}^{0.5}$$

where $A_{k}$ and $B_{k}$ are correction constants for the gas to be measured after calibration. This is the most commonly used formula for classic convection heat transfer. On this basis, Kramers proposed a more general heat transfer formula [10]:

(6) $$N_{u}=0.42{P_{r}}^{0.2}+0.57{P_{r}}^{0.33}\cdot {R_{e}}^{0.5}.$$

In summary, we have

(7) $$H=\pi l\lambda_{f}\Delta T\left(0.42{P_{r}}^{0.2}+0.57{P_{r}}^{0.33}{R_{e}}^{0.5}\right)$$

where $A_{c}=0.42\pi l\lambda_{f}{P_{r}}^{0.2}$ and $B_{c}=0.57\pi l\lambda_{f}{P_{r}}^{0.33}$. $A_{c}$ and $B_{c}$ are regarded as constants, which are determined by the size of the metal sensing head, the gas properties and the flow conditions. With Equation (7), one obtains

(8) $$H=(A_{c}+B_{c}\cdot {R_{e}}^{0.5})\cdot \Delta T.$$

Roger Baker has given another expression of hA:

(9) $$hA=hld\pi =A_{c}+B_{c}{\left(q_{m}\right)}^{0.5}$$

where $q_{m}$ means the mass of the flow. Finally, one can obtain

(10) $$q_{m}={\left[\frac{1}{B_{c}}\cdot \frac{H}{\Delta T}-\frac{A_{c}}{B_{c}}\right]}^{2}.$$

From the equation above, we can clearly see a relation between the mass of flow $q_{m}$ and the temperature difference ΔT. The greater the $q_{m}$, the smaller the ΔT.

### 2.3. FBG Temperature Measurement Method

According to the principle of heat flow sensor, temperature of the gas and the thermal dissipation need to be measured in real time. We could use FBG to sense the temperature.

For an FBG, ambient temperature can exert effect through shift of the wavelength of Bragg $\lambda_{B}$ caused by thermal-expansion effect and thermal-optic effect. From the Bragg conditions, we have

(11) $$\lambda_{B}=2n_{eff}\Lambda$$

where $n_{eff}$ and Λ represent the effective refractive index and grating period of the FBG. The Bragg wavelength changes with the effective refractive index and the grating period. Take the natural logarithm of Equation (11) and derive the derivative for temperature *T*:

(12) $$\frac{1}{\lambda_{B}}\cdot \frac{d\lambda_{B}}{dT}=\frac{1}{n_{eff}}\cdot \frac{dn_{eff}}{dT}+\frac{1}{\Lambda }\cdot \frac{d\Lambda }{dT}$$

where the first term on the right side is related to the thermal-optic effect, which results in the change in refractive index, while the second term is concerned with the thermal-expansion effect that causes the change in the grating period.

For the quartz fiber, in room temperature, the thermal expansion coefficient is 0.5 × 10^−6^/°C, the thermal-optic coefficient is 8.3 × 10^−6^/°C [11]. The temperature sensitivity of FBG at 1550 nm can be estimated as 13 pm/°C. It should be emphasized that this theoretical value is calculated by the parameters for quartz material. While in the process of manufacturing optical fiber and writing fiber grating, some elements like Ge are doped into the fiber core, which results in a tiny change in the above parameter value. Therefore, the temperature sensitivity of real FBG is different from the estimated value.

## 3. Design of Heat Flow Sensor

**Figure 2:**
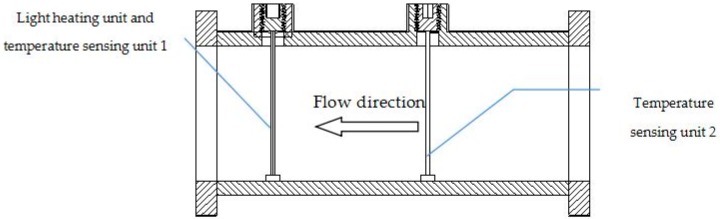
Optical thermal gas flow sensor.

The overall design of the optical heat flow sensor includes the pipe between the flanges with the light-heating and two temperature-sensing units, as shown in Figure 2.

**Figure 3:**
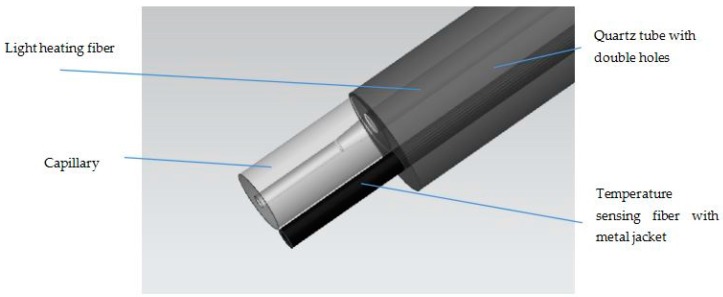
Self heating and temperature sensing head.

In order to prevent the explosion caused by electrical spark, we adopt a light heating module, in which one optical fiber as the heating channel transfers the energy. The other fiber is used for sensing temperature and is jacketed by a metal tube. It should be pointed out that one end of the fiber is fixed in the metal tube, while the other end is freely stretchable, and the FBG is non-strain packaged, as seen in Figure 3. In fact, in the real fabrication, the fiber diameter is a little less than the inner diameter of the metal tube, which guarantees that the fiber and steel tube could not tightly embrace together in the operation range. 

In order to prevent the interference between fibers, we employ a double-hole quartz tube to isolate the two fibers. The light from the heating fiber inject to a capillary with a tapered structure, which is connected to quartz tube by using optical glue. The light is absorbed by the film coated on the surface of the capillary and turned into heat energy.

Metal jacket can protect the grating from the influence of vibration and stress of the pipe, and steel has high temperature sensitivity, since the coefficient of thermal expansion of metal is much larger than that of the optical fiber. The capillary is tightly bonded to the metal jacket. 

**Figure 4:**
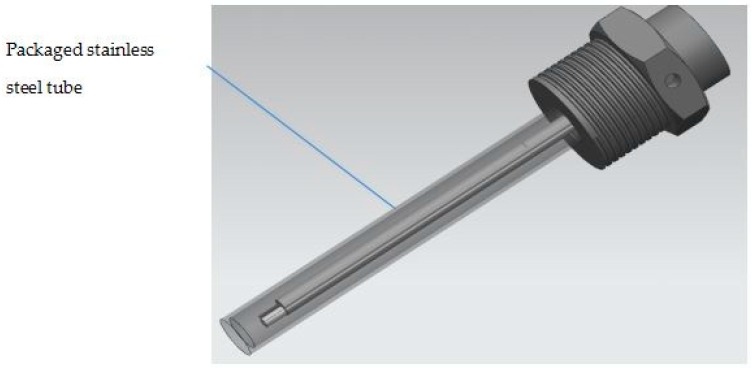
Integrated light heating and temperature sensing unit.

**Figure 5:**
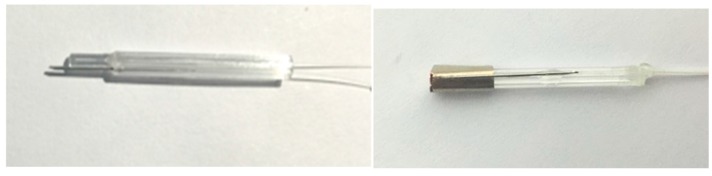
The real picture of sensor units.

In the real cases, we have selected light absorption coating with high conversion efficiency in C band. The absorption ratio of coating in the wavelength range of 0.4~2 um is greater than 0.9. The integrated unit with optical-heating and temperature-sensing is as shown in Figure 4. The self heating and temperature sensing unit is packaged in a stainless steel tube. Figure 5 gives a real picture of sensor units corresponding to Figure 4. 

## 4. Experiment and Analysis

In order to investigate the linearity of temperature-sensing unit, we use a constant temperature box to calibrate the parameters of temperature-sensing unit. This unit adopts the grating, whose specific parameters are listed in Table 1.

**Figure 6:**
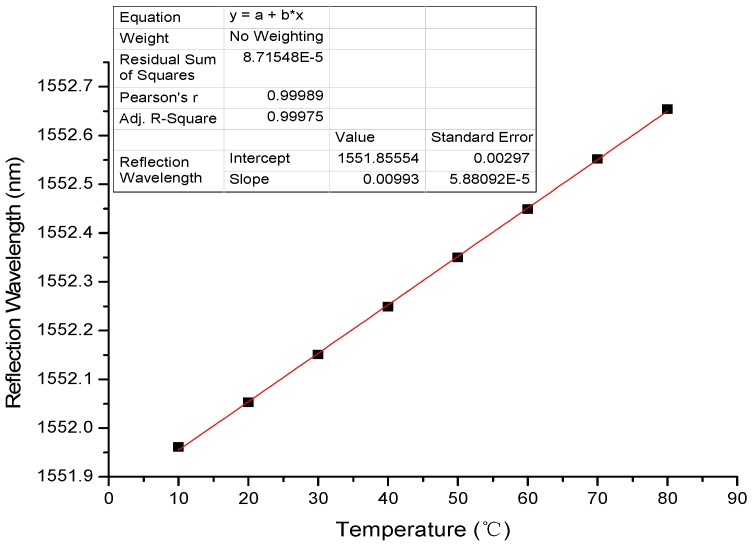
Calibration curve of fiber Bragg grating (FBG) temperature sensor.

The measured dependence of wavelength shift on temperature is shown in Figure 6. From the figure, one can easily estimate the temperature sensitivity of the sensor near 10 pm/°C. Obviously, the value of sensitivity is lower than that estimated before. Except the reason stated before in Section 2.3, there is another reason to explain this phenomenon that the real sensor is composed of not only the neat FBG but also the metal jacket. The reduction of sensitivity is natural. 

According to the wavelength of reflected light, the corresponding temperature may be obtained, which meets the experimental requirements. This dependence can cover the whole temperature range used in the flow sensor.

**Figure 7:**
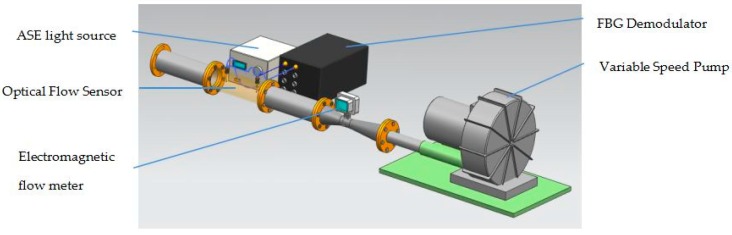
Diagram of the test system.

Our sensor is plugged in a segment of real test pipe, please see Figure 7. The gas velocity, which is monitored by a gas electromagnetic flow meter, can be adjusted by a variable speed pump. The light in C band from an ASE light source, 80% of which is provided to the light heating unit and at the same time the rest (20%) of which is used for the temperature sensing units to detect the temperature.

The signals on temperature are demodulated by an FBG demodulator. The demodulator achieves wavelength demodulation using wavelength selective characteristics of the Fabry-Perot (F-P) cavity. The narrow-band spectrum reflected by the sensing grating enters the F-P filter through the coupler, when the reflection spectrum of the sensing grating coincides with the transmission spectrum of the filter, the output power of the filter is maximum. The transmission spectrum is related to the spacing of the F-P cavities, the spacing of the F-P cavities is controlled by the scan voltage on the piezoelectric. Therefore, the wavelength can be demodulated by measuring the scanning voltage corresponding to the maximum light intensity value [12].

The parameters of the ASE light source and the demodulator are listed in Table 2 and Table 3.

**Figure 8:**
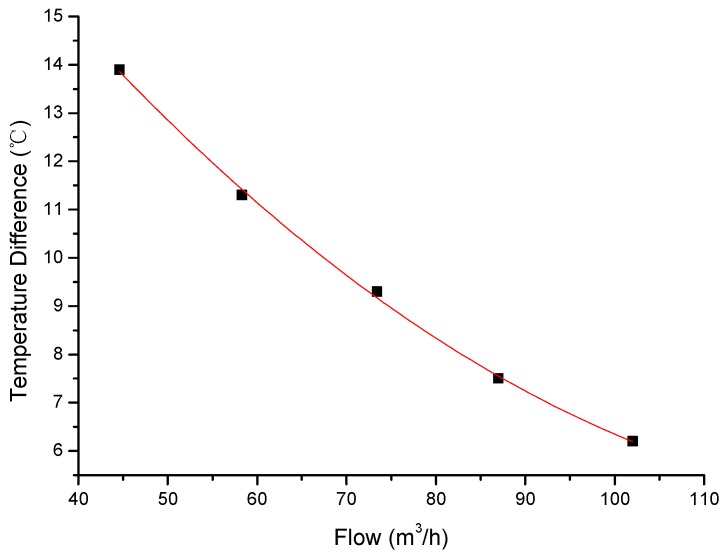
The fitting curve of flow-temperature difference.

We test the relation between the flow and temperature differences, reflected by the central wavelength shift of two FBG temperature units, and obtain a curve of Δ*T* vs. *q_m_*, as shown in Figure 8. From the curve, one can see that the behaviors of *q_m_* with Δ*T* are well consistent with our theory prediction. It should be pointed out that, in this curve, in the lower flow range, the temperature difference is big enough for measurement resolution, while, in the higher flow range, the difference become smaller, which may result in a decrease in sensitivity of measurement. In practice, one should enhance the heating power to solve this problem. In the real test, the following factors should be considered which may influence the reliability: one is the resolution of the demodulation and the bandwidth of the FBG, another one is the accuracy of calibrated flow meter and the fluctuation of the flow should also be controlled. 

When the temperature difference is decreased by 1 °C, the flow value changes 7.7 m^3^/h, the resolution of the demodulator is 1 pm, that is 1 °C, the volumetric resolution is 0.77 m^3^/h, and the velocity resolution is 0.04 m/s. 

## 5. Conclusions

In summary, we proposed an FBG-based sensor for measuring the heat flow of gas which has the advantage of anti-electromagnetic interference ability and reliable structure. The test results demonstrate good consistence with the simulation. The special design of the heating head with light may guarantee the sensor to be explosion-proof in gas measurement, which is meaningful for the practical applications. The method for improving the measurement accuracy was also discussed. 

## Figures and Tables

**Table 1:** Parameters of the grating.

Parameters	Value
Central wavelength	1550 nm
Length of grating region	10 mm
3 dB bandwidth	≤0.3 nm
Reflectivity	≥80%

**Table 2:** Parameters of ASE light source.

Parameters	Value
Operating wavelength	1525~1565 nm
Output Power	10~17 dBm
Spectral density	≥−20 dBm/nm
Spectral flatness	≤2 dB
Fiber type	Corning SMF−28
Fiber output end type	FC/APC

**Table 3:** Parameters of FBG demodulator.

Parameters	Value
Wavelength range	1525~1565 nm
Wavelength resolution	±1 pm
